# Effect of apple cider vinegar on delayed gastric emptying in patients with type 1 diabetes mellitus: a pilot study

**DOI:** 10.1186/1471-230X-7-46

**Published:** 2007-12-20

**Authors:** Joanna Hlebowicz, Gassan Darwiche, Ola Björgell, Lars-Olof Almér

**Affiliations:** 1 Department of Medicine, University of Lund, Malmö University Hospital, Malmö, Sweden; 2 Department of Radiology, University of Lund, Malmö University Hospital, Malmö, Sweden

## Abstract

**Background:**

Previous studies on healthy people show that vinegar delays gastric emptying and lowers postprandial blood glucose and insulin levels. The aim of this study was to investigate the effect of apple cider vinegar on delayed gastric emptying rate on diabetes mellitus patients.

**Methods:**

Ten patients with type 1 diabetes and diabetic gastroparesis, including one patient who had undergone vagotomy, were included and completed the investigator blinded crossover trial. The gastric emptying rate (GER) was measured using standardized real-time ultrasonography. The GER was calculated as the percentage change in the antral cross-sectional area 15 and 90 minutes after ingestion of 300 g rice pudding and 200 ml water (GER1), or 300 g rice pudding and 200 ml water with 30 ml apple cider vinegar (GER2). The subjects drank 200 ml water daily before breakfast one week before the measurement of GER1. The same subjects drank 200 ml water with 30 ml vinegar daily before breakfast for two weeks before the measurement of GER2.

**Results:**

The median values of GER1 and GER2 were 27% and 17%, respectively. The effect of vinegar on the rate of gastric emptying was statistically significant (p < 0.05).

**Conclusion:**

This study shows that vinegar affects insulin-dependent diabetes mellitus patients with diabetic gastroparesis by reducing the gastric emptying rate even further, and this might be a disadvantage regarding to their glycaemic control.

**Trial registration number:**

ISRCTN33841495.

## Background

Diabetes mellitus is a growing problem globally. According to recent estimates there were over 171 million people living with diabetes worldwide in 2000, and the number is estimated to increase to 366 million by 2030 [1]. Studies have shown that 30–50% of diabetes patients have delayed gastric emptying and this is believed to be, at least partially, due to vagal denervation caused by autonomic neuropathy [2-6]. Delayed gastric emptying may cause poor glycaemic control, especially in those with preprandial antidiabetic treatment leading to/causing postprandial hypoglycaemia and gastrointestinal symptoms such as postprandial nausea, vomiting, bloating and early satiety [7]. The relationship between the symptoms of gastroparesis and the rate of gastric emptying is weak, and patients with delayed gastric emptying may not have any, or few, gastrointestinal symptoms [8-10]. An increased frequency of hypoglycaemic events in insulin-treated diabetic patients has been associated with abnormal gastric emptying, despite the lack of upper gastrointestinal symptoms [11]. However, diabetes mellitus has been associated with an increased prevalence of upper and lower gastrointestinal symptoms compared with healthy subjects, and these symptoms have been found to be associated with poor glycaemic control but not the duration of diabetes [12]. There is a significant, albeit weak, relationship between gastric emptying of solids and the presence of upper gastrointestinal symtoms; increased retention in the distal but not proximal stomach is associated with increased gastrointestinal [13].

Studies have shown that vinegar reduces postprandial blood glucose levels in healthy subjects [14-17], and it has been discussed whether this could be explained by delayed gastric emptying [14,15]. However, the effect of vinegar on gastric emptying in diabetic patients with gastroparesis has not been studied previously. In a recent study, apple cider vinegar was shown to improve insulin sensitivity, and lowered the postprandial blood glucose and insulin levels in insulin-resistant subjects [18]. Subjects with type 2 diabetes showed a slight improvement in insulin sensitivity, but postprandial blood glucose and insulin levels were not affected when apple cider vinegar was added to a meal [18]. In the Framingham offspring study a diet with high glycaemic index was found to be associated with metabolic syndrome [19]. A diet with low glycaemic index, such as a meal including vinegar, is favourable in healthy subjects and in insulin-resistant subjects [14-18]. In Sweden, and in other European countries, it is the custom to consume vinegar in salad dressing. It is common for patients with diabetes in Sweden to drink vinegar daily because of its positive effect on blood glucose. However, if vinegar delays the gastric emptying rate (GER), this may cause instability of the metabolic control and increase gastrointestinal symptoms or the frequency of hypoglycaemic events in insulin-dependent diabetes mellitus patients with gastroparesis, especially those injecting insulin preprandially. Insulin-dependent diabetes patients should perhaps also take into account the composition of the meal and its affects on the GER, as well as the carbohydrate content of the meal before the administration of short-acting insulin.

The aim of this study was therefore to evaluate the influence of vinegar on the GER in insulin-dependent diabetes mellitus patients with diabetic gastroparesis.

## Methods

Ten patients with type 1 diabetes mellitus and with diagnosed diabetic gastroparesis (five men and five women; mean age 67.9 ± 8.0 years [range 57–79 years]; mean Body Mass Index 25.4 ± 2.9 kg/m^2 ^[range 21.2–30.9 kg/m^2^]; mean duration of diabetes 41.3 ± 13.2 years [range 18–57 years], mean value of Hemoglobin A1c 8.3 ± 0.7 % [range 7.5–9.5]) were included in and completed the crossover study. Patients were recruited from those previously diagnosed with gastroparesis measured by a previously described and standardised scintigraphic and real-time ultrasound method [20] at the Malmö University Hospital. A GER lower than 45% indicates delayed gastric emptying, and has been shown to be strongly correlated to scintigraphic half-time values [21]. The patients had symptoms typical of diabetic gastroparesis (postprandial abdominal fullness or nausea, vomiting, postprandial early satiety or early postprandial hypoglycaemia, despite the ingestion of food and correctly taken doses of insulin).

Seven of the subjects had a history peripheral neuropathy, eight had retinopathy and four had nephropathy. Those with renal failure (microalbuminuria > 20 microg/min), previously major abdominal surgery, history of severe cardiovascular disease and hepatic disease were excluded from the study. The patients showed no evidence of prior gastric outlet obstruction or connective tissue diseases. One of the patients had had his gall bladder removed, and two patients had had their uterus and ovaries removed, three patients had been appendectomized, and one had undergone vagotomy 16 years previously. All of the patients were being treated with a multiple-dose regime, consisting of rapid- or short-acting insulin before meals and intermediate-acting insulin once or twice daily. No medication was changed, and no subject used any medication with known major gastrointestinal side effects during the study. None of the subjects used any prokinetic treatment before or during the study. Two of the subjects were snuff users but none smoked; snuff-taking was prohibited for 8 h before and during the test. The subjects were examined in the morning between 8:00 and 10:00 after fasting for 8 hours. The examination was conducted providing that the subject's fasting blood glucose level was in the range 3.5–9.0 mmol/l. The subjects were asked not to consume any drugs or insulin on the day of the examination. If the blood glucose level was lower than 3.5 mmol/l or higher than 9.0 mmol/l on the day of the study the examination was postponed. Blood glucose concentrations were measured with the HemoCue Glucose system (HemoCue AB, Ängelholm, Sweden). Before each meal the subjects took their normal daily insulin dose which was not changed during the study. If the subjects reported gastrointestinal symptoms (diarrhoea, constipation, nausea or vomiting) on the day of the study the examination was postponed. Patients with chronic constipation (in our study defined as symptoms for 1 year or longer) were not excluded, as this was assumed to be their basal state, possibly owing to autonomic neuropathy. GER measurements were made on two different occasions.

One week before the first examination, the patients drank 200 ml water every morning before their breakfast. The reference meal consisted of 300 g rice pudding (BOB, Scan Foods AB, Johanneshov, Sweden, 100 g caloric value 110 kilocalories, 3.5 g protein, 17 g carbohydrates and 3 g fat). GER measurements were then made after drinking 200 ml water before the ingestion of the rice pudding. Each meal had to be consumed within 10 min and the water was consumed before ingestion of the rice pudding meal. After the first examination the patients were given a 450 ml bottle of commercial available apple cider vinegar with honey flavouring (Druvan, DR Persfood AB, Eslöv, Sweden). The caloric value of 100 ml apple cider vinegar was 65 kilocalories, 16 g carbohydrates, < 0.5 g protein and 0 g fat. The apple cider vinegar was composed of 5% acetic acid and had a pH value of 2.8–3. The patients drank 200 ml water mixed with 30 ml apple cider vinegar (total volume 230 ml) every morning for two weeks before their breakfast. GER measurements were then made after drinking 30 ml apple cider vinegar mixed with 200 ml water (total volume 230 ml) before the ingestion of the rice pudding. Each meal had to be consumed within 10 min and the water, with vinegar, was consumed before ingestion of the rice pudding meal. One of the subjects who participated in the study was already consuming apple cider vinegar daily, and was therefore examined for the first time after drinking apple cider vinegar mixed with water before ingestion of the rice pudding. After a ten-day wash-out period with water before the ingestion of breakfast, the same subject was examined after the ingestion of the reference meal. The subjects were not prohibited to consume any other vinegar or acetic-acid containing products during the study. The order of the two different meals were not randomized.

Gastric emptying was measured using ultrasonography employing a previously described method [20]. We did not have a specific room with only one machine and we used the available ultrasound machine at the moment. The sonographic examination was performed using four different ultrasound machines ((Acusone Sequioa 512, Mountain View, CA), (Aloka ProSound SSD 5500, Tokyo, Japan), (Siemens Elegra, Siemens Medical Solutions, Mountain View, CA), (B-K Medical 2102 Hawk, Gentofte, Denmark)) with an abdominal 2.5–4 MHz transducer. For each calculation of the GER, the antrum diameters had been measured using only one of the above machines. However, the same ultrasound machine was not used for the paired studies. Measurements of the gastric antrum were performed by the same radiologist who was blinded with regard to the meals. The radiologist applied minimal abdominal compression with the abdominal transducer during the measurements. The subjects were examined in the supine position, but sat between the examinations, 15 and 90 minutes after the end of meal ingestion. The GER was expressed as the percentage change in the antral cross-sectional area from 15 to 90 min. Three measurements were made of the longitudinal (d_1_) and anteroposterior (d_2_) diameters at each examination, and the mean value was used to calculate the cross-sectional area of the gastric antrum using the following formula:

Antrum area = π × r^2 ^= π × d_1_/2 × d_2_/2 = π × [d_1 _× d_2_]/4

At each measurement of the gastric antrum the abdominal aorta and the left lobe of the liver were used as internal landmarks. The GER was calculated using the following formula:

GER = [1- (Antrum area 90 min/Antrum area 15 min)] × 100

The study was performed according to the Helsinki declaration. All subjects gave written, informed consent before participating in the experiments.

Median values with quartiles (q1 to q3) are presented for the antral cross-sectional areas and the GER. All statistical calculations were performed in SPSS for Windows (SPSS (Version 14.0, 2005;Chicago IL, USA). The significance of differences in GER, antral cross-sectional areas and blood glucoe values were evaluated with the Wilcoxon t-test. P-values < 0.05 were considered significant.

## Results

### Blood glucose

The mean fasting blood glucose levels were before the reference meal 6.9 ± 0.6 mmol/l compared and not significant different to before ingestion of the meal including vinegar 7.3 ± 0.5 mmol/l.

### GER

**Figure 1:**
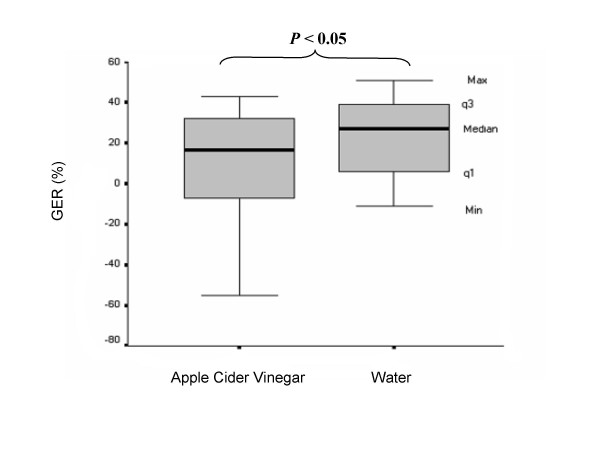
Gastric emptying of a rice pudding meal ingested with and without apple cider vinegar, expressed as the gastric emptying rate (GER), in ten type 1 diabetics with clinically diagnosed diabetic gastroparesis. The median (Md), minimum (Min), and maximum (Max) values and the values of the first (q1) and the third (q3) quartiles are shown. Values of p < 0.05 were considered significant.

The median values of the antral cross-sectional area after ingestion of the meal including vinegar were 887.5 mm^2 ^(range 654 to 1626 mm^2^, q1 = 694 mm^2^, q3 = 1230 mm^2^) and 786 mm^2 ^(range 447 to 1851 mm^2^, q1 = 586 mm^2^, q3 = 959 mm^2^) at 15 and 90 min, respectively, after the end of the meal, compared to 866 mm^2 ^(range, 602 to 1710 mm^2^, q1 = 725 mm^2^, q3 = 1071 mm^2^) and 611 mm^2 ^(range, 295 to 1709 mm^2^, q1 = 561 mm^2^, q3 = 760 mm^2^) at 15 and 90 min, respectively, after the end of the reference meal. The median gastric antral cross-sectional areas were significantly larger after ingestion of the meal including vinegar than after the reference meal including water at 90 min (p < 0.05), but there were no significant differences between gastric antral cross-sectional areas at 15 min. The median value of the GER after the meal including vinegar was 17% (range -55% to 43%, q1 = -9%, q3 = 32%), while the median value of the GER after the reference meal 27% (range -11% to 51%, q1 = 5%, q3 = 41%). These results are shown in Figure 1. Gastric emptying rates after the meal including vinegar were significantly lower (p < 0.05) than after the reference meal. Individual values of GER indicated reduced values in all patients except two, after drinking apple cider vinegar (Figure 1).

## Discussion

The primary objective of this pilot study was to determine weather apple cider vinegar would improve a delayed gastric emptying rate on diabetes mellitus patients. Despite the fact that we only studied ten patients with type 1 diabetes mellitus and diabetic gastroparesis, we were able to demonstrate a significant delay in already delayed gastric emptying of these subjects after the ingestion of vinegar. The median gastric antral cross-sectional areas were significantly larger after ingestion of the meal including vinegar than after the reference meal at 90 min. This difference could be due to delayed gastric emptying and therefore increased amount of gastric juices and saliva in the stomach. The subject that had undergone vagotomy responded in the same way as the others, showing a further reduction in the GER after drinking apple cider vinegar. The subject that was already consuming vinegar daily before of the start of the study also showed a reduced GER after drinking apple cider vinegar. He reported fewer gastrointestinal symptoms after the consumption of vinegar for a long period, which prompted us to initiate this study.

We hypothesized that daily consumption of vinegar would affect diabetes patients with gastroparesis by increasing the gastric emptying rate. However, the effect found was the opposite in this study, namely the same as that observed by others in healthy subjects [15], i.e. a decrease in the rate of gastric emptying after the intake of vinegar. One of the subjects who already had a slow GER spontaneously reported a higher frequency of hypoglycaemic episodes during the two-week period of drinking apple cider vinegar. Neither the severity of the symptoms nor the frequency of hypoglycaemic episodes was recorded in this study. However, poor correlation has been observed between gastrointestinal tract symptoms and gastric autonomic neuropathy among subjects with diabetes mellitus [8]. Bloating and fullness have been associated with diabetic gastroparesis, whereas other upper gastrointestinal symptoms were found to be correlated weakly with solids emptying, and not at all with liquid emptying [9]. An increase in the frequency of unexplained hypoglycaemic episodes has, however, been found to be associated with an abnormal GER [11]. The measurements of gastric emptying were performed by the same radiologist who was blinded with regard to the meals. However, a limitation of this study is that it was not randomized and the subjects were not blinded. Another limitation of this study is that there may be a variation in measurments between the different machines used in the paired studies.

In a recent study, it was shown that white wheat bread served with white vinegar, reduced the postprandial blood glucose and insulin levels, and not only increased but also prolonged satiety in healthy subjects [17]. A dose-response relationship was also found between the amount of vinegar added and the levels of glucose, insulin and satiety [17]. Vinaigrette sauce added to cold boiled potatoes has also been found to reduce the postprandial blood glucose and insulin levels in healthy subjects [16]. The mechanism by which vinegar reduces postprandial blood glucose levels has been suggested to be a delay in gastric emptying [15], or the inhibition of amylases [14]. However, the organic acid sodium propionate has not been shown to affect starch hydrolysis *in vitro *[22]. In both healthy subjects and patients with type 1 diabetes mellitus it has been shown that hypoglycaemia increases the rate of gastric emptying [23,24]. Hyperglycaemia has also been shown to decrease gastric emptying in insulin-dependent diabetes mellitus patients [25]. Even physiological changes in blood glucose, from 4 to 8 mmol/l have been shown to affect gastric emptying in healthy subjects and in insulin-dependent diabetes mellitus patients [26]. Unfortunately, did we not measure the postprandial blood glucose levels. However, the examination was performed only if the fasting blood glucose level was in the range 3.5 to 9.0 mmol/l and none of the subjects reported any symptoms of hypoglycaemia on the day of the ultrasonography examinations. The mean fasting blood glucose level before the reference meal was not significantly different from that before ingestion of the meal including vinegar.

It has been shown, using paracetamol as a marker for gastric emptying, that gastric emptying is delayed in healthy subjects after a meal including white vinegar [15]. The paracetamol method is dependent on the absorption of paracetamol across the small intestine which makes this method unreliable, as the pharmacokinetics of paracetamol vary within and between individuals [27,28]. The antihyperglycaemic effect of acetic acid has been shown to be mediated by enhanced glycogen repletion in liver and skeletal muscle [29], and in the suppression of disaccharidase activity in human intestinal cells [30]. In insulin-resistant subjects, apple cider vinegar has also been shown to improve postprandial insulin sensitivity [18]. It has been suggested that non-specific acid or pH receptors in the small intestine could reduce the GER [31]. The postprandial blood glucose level in healthy subjects was found to be reduced after a meal including white vinegar, compared with a meal including neutralised vinegar [14]. However, in the same study, gastric emptying measured using ultrasonography showed no difference in antral area after the reference meal compared with the meals including white vinegar or neutralised white vinegar [14].

Whether low-glycemic-index food in fact prevents diabetes mellitus is still unclear [32-36]. However, a low-glycemic-index diet that reduces postprandial hyperglycemia is recommended by the American Diabetes Association (ADA) to control glycemia in patients with diabetes [37,38]. Therefore the benefit findings of vinegar that has been shown to improve postprandial insulin sensitivity in insulin-resistant subjects [18] needs to be further studied. However, if vinegar reduces postprandial hyperglycemia and delays gastric emptying, the doses of preprandial injections of short-acting insulin may have to be adjusted. Furthermore, blood glucose must be monitored more frequently in patients with diabetes treated with insulin experiencing gastroparesis to prevent adverse hypoglycaemic episodes.

## Conclusion

This small study show that vinegar delays gastric emptying in insulin-dependent diabetes mellitus patients with diabetic gastroparesis. Clearly, a larger, randomized trial involving a greater number of patients would be needed to validate the findings of this pilot study.

## Competing interests

The author(s) declare that they have no competing interests.

## Authors' contributions

JH participated in the design of the study, recruited the subjects, performed the statistical calculations and drafted the manuscript. GD participated in the design of the study, and participated in drafting the manuscript. OB participated in the design of the study and performed the ultrasound examinations. LOA participated in the design of the study and drafting of the manuscript. All authors read and approved the final manuscript.

## Pre-publication history

The pre-publication history for this paper can be accessed here:


